# Supplementary material for: Native nucleosomes intrinsically encode genome organization principles
Source: Nature. 2025 May 7;643(8071):572–81. doi: 10.1038/s41586-025-08971-7 (PMC12240700; doi:10.1038/s41586-025-08971-7)
Supplement: Supplementary file 1 — Supplementary Notes 1-4, Supplementary Tables 1-12, Supplementary Fig. 1 and Supplementary References. [file 41586_2025_8971_MOESM1_ESM.pdf]

nature portfolio

https://doi.org/10.1038/s41586-025-08971-7

Supplementary information

Native nucleosomes intrinsically encode genome organization principles

In the format provided by the authors and unedited

Supplementary Note 1. Native mono-nucleosome purification protocol.

Mammalian cell culture

For the H1-hESC cell culture, we followed the 4D Nucleome standard protocol[1]. In brief, H1-hESC cells were cultured on Matrigel (Corning Matrigel hESC-Qualified Matrix, LDEV-free) coated 150x21mm Petri dishes with mTeSR1 (STEMCELL technologies) medium, which was exchanged daily. The H1-hESC cells were cultured until 70-80% confluency before harvesting. For GM12878 cell culture, we also followed the standard protocol from the 4D Nucleome portal[1]. E14 mouse ESC were cultured in DMEM (Corning, cat no. 10-013-CV) containing 10% fetal calf serum (Corning, cat no. 35-011-CV), 100 Units/ml leukemia inhibitory factor (Millipore, cat no. ESG1107), 1×MEM non-essential amino acids (Invitrogen cat.no. 11140050) and 100 mM 2-mercaptoethanol (Gibco cat no. 21985-023). Cells were cultured on 0.1% gelatin (Sigma Aldrich, cat no. G1393-100ml) coated tissue culture flasks.

Nuclei isolation

We adopted the previous hydroxyapatite (HAP) purification protocol[2] for nucleosome purification. First, we harvested about ~100 million cells using trypsin and spun them at 520 g for 5 min at 4 °C. The cell pellets were resuspended in cold PBS with protease/deacetylase/phosphatase inhibitors (400 μM PMSF, 1 μM Trichostatin A, 10 mM β-Glycerophosphate, 2 tablets/100 ml cOmplete, Mini EDTA-free protease inhibitor tablet) and then we spun down the cells (repeat this step twice). The cells were resuspended in the cold Buffer N (15 mM Tris-HCl pH 7.5, 15 mM NaCl, 60 mM KCl, 250 mM sucrose, 5 mM MgCl2, 1 mM CaCl2 at the same concentration of protease/deacetylase/phosphatase inhibitors and 1 mM DTT) and were spun down (repeat this step twice). The cell pellets were then resuspended with 1ml Buffer N and 1 ml of 2x lysis buffer (Buffer N with 0.6% NP-40) was added dropwise. The suspension was incubated on ice for 10 min to complete cell lysis. The isolated nuclei were then spun down at 600 g for 8 min at 4 °C. The nuclei were then resuspended in Buffer N and spun down two more times. Finally, the nuclei were resuspended in 500 μl of ice-cold Buffer N. The yield of the isolated nuclei was checked using UV-VIS spectrometer at the 260 nm absorbance.

In-nuclei MNase digestion of chromatin

MNase (Worthington Biochemical Corporation) was added to the nuclei solution and incubated at 37 °C for 10 min. The working concentration of MNase was adjusted to optimize the yield of mono-nucleosomes, while avoiding over-digestion. The reaction was stopped by adding a 1/10 volume of MNase stop buffer (110 mM EDTA, 110 mM EGTA). Then, 0.5% Triton-X 1/10-volume was added to MNased sample (final 0.05% Triton-X), and the nuclei debris was removed by centrifuge at 15,000 g for 5 min at 4 °C. The supernatant that contained the released MNase-digested chromatin was taken.

HAP purification of nucleosomes

The HAP slurry was prepared by mixing 200 μl HAP buffer 1 (3.42 mM Na_2HPO_4, 5 mM NaH_2PO_4, 600 mM NaCl, 1 mM EDTA at the same concentration of protease/deacetylase/phosphatase inhibitors as in nuclear isolation) and 66 mg of hydroxyapatite resin (Macro-prep ceramic hydroxyapatite type I, 20 μm, Bio-Rad) per 100 μg of chromatin at the weight of DNA and mixed together with the chromatin. The HAP mix was vortexed and incubated in a rotator at 4 °C for 10 min. The HAP mix was transferred to a spin column (Pierce™ Micro-Spin Columns) and centrifuged for 1 min at 600 g at 4 °C. The flow-through was discarded or saved for quality control of the purification steps. Then, 200 μl of HAP buffer 1 was added to the column and centrifuged under the same condition. Next, 200 μl of HAP buffer 2 was added to the column and centrifuged for 1 min at 600 g in 4 °C. The flow-through was discarded or saved for quality control (repeat five times). Then,100 μl of HAP elution buffer was added to the column and centrifuged for 1 min at 600 g at 4 °C (repeat three times). The elution fractions were saved as the HAP purified nucleosomes. The yield of the HAP purified nucleosomes was checked using UV-VIS spectrometer at 260 nm absorbance. For quality control of the HAP purification steps, the DNA from the HAP flow-through and elution fractions was purified by phenol-chloroform extraction and was run in 2% agarose gel to check the ladder-like DNA banding pattern for evidence of MNase-treated chromatin, which is only shown in the elution fractions, not flow-throughs.

Size-selective purification of mono-nucleosomes

Although HAP elution products are mostly mono-nucleosomes, they include a mixture of mono-nucleosomes, oligo-nucleosomes, and naked DNA. A further size-selective purification was performed using a PAGE gel-based column (Bio-Rad Mini Prep Cell) to obtain purer mono-nucleosomes. The HAP elution sample was applied to a 6–7% PAGE gel column and eluted into different size fractions, while the gel was run at a constant 1 W for 3–5 hours at 4 °C. Each fraction was run in 6% PAGE gel to check its corresponding size and to compare it to the input. The fractions were carefully chosen based on the similar migration of the major HAP elution gel band, which consists mainly of mono-nucleosomes. After this step, only ~10% of the total HAP elution was purified into more a pure mono-nucleosome sample. The quality of the final purified nucleosome sample was validated by running it in 2% agarose gel, 6% PAGE gel, SDS-PAGE with Coomassie staining, and western blotting for various histone PTM markers.

Solubilization of spermine condensed nucleosomes

Nucleosomes (native GM12878, reconstituted GM12878 and native E14 mouse ESC) were dialyzed into 10mM Tris pH 7.5 buffer through three buffer exchanges using an Amicon Ultra 10kD filter (MilliporeSigma). The final concentration of nucleosomes for each condensation reaction was 50ng/ul (determined as DNA weight). The condensation buffer contained 10mM Tris pH7.5 with 50mM NaCl and 0.2mg/ml BSA. Two reactions using 0mM and 0.5mM spermine were simultaneously prepared. The condensation assay was performed as previously explained. After centrifugation, the pellet was resuspended in 12ul of 10mM Tris buffer pH7.5 and incubated for 10 min at room temperature, remixing the suspension every two minutes. Solubilized nucleosomes were checked by running a 6% Native PAGE gel at 4°C. Solubilized reconstituted nucleosomes were directly loaded to the PAGE gel by taking 1.5ul of the solution. For Native

GM12878 and Native E14 mouse ESC, 8ul were first removed from the suspension and the remaining volume was thoroughly mixed immediately before loading to the PAGE gel.

Supplementary Note 2. Pair-wise interaction energy calculation and chromatin polymer simulation.

Chromatin was modeled as a beads-on-a-string polymer. Each bead represents a 25-kilobase-long genomic segment. The energy function of chromatin is defined as follows:

$$U_{\text{simulation}}(\mathbf{r}) = U_{\text{bond}} + U_{\text{EV}} + U_{\text{confine}} + U_{\text{contact}}.$$

The first term establishes connectivity between the nearest neighbors. It adopts the following functional form

$$U_{\text{bond}} = \sum_{i=1}^{N-1} k_2 \left(r_{i,i+1} - r_0\right)^2 + k_3\left(r_{i,i+1} - r_0\right)^3 + k_4\left(r_{i,i+1} - r_0\right)^4,$$

where i is the indices over the chromatin beads and N is the total number of beads. The value r_0 in this expression is the equilibrium bond length which is set to bead diameter σ (i.e. the beads are touching each other at equilibrium), and $k_2 = 20\frac{k_B T}{\sigma^2}$, $k_3 = 20\frac{k_B T}{\sigma^3}$, and $k_4 = 20\frac{k_B T}{\sigma^4}$ are global parameters that define the bond strength.

$U_{\text{EV}} = \sum_{i>j}^{N} U_{\text{sc}}\left(r_{ij}\right)$ accounts for the excluded volume effect and $U_{\text{sc}}\left(r_{ij}\right)$ is defined as follows:

$$U_{\text{sc}}\left(r_{ij}\right) = \begin{cases} 0.5E_{\text{cut}}\left(1 + \tanh\left(\frac{2U_{\text{WCA}}\left(r_{ij}\right)}{E_{\text{cut}}}\right)\right), & r_{ij} < r_S \\ U_{\text{WCA}}\left(r_{ij}\right), & r_S < r_{ij} < 2^{\frac{1}{6}}\sigma \\ 0, & r_{ij} > 2^{\frac{1}{6}}\sigma \end{cases}$$

With this potential, we defined U_{WCA}, r_S and E_{cut} as follows

$$U_{\text{WCA}}\left(r_{ij}\right) = 4k_B T\left[\left(\frac{\sigma}{r_{ij}}\right)^{12} - \left(\frac{\sigma}{r_{ij}}\right)^{6}\right] + k_B T, \qquad r_S = 2^{\frac{1}{6}}\sigma\left(\sqrt{\frac{E_{\text{cut}}}{2}} + 1\right)^{\frac{-1}{6}}, \qquad E_{\text{cut}} = 4k_B T.$$

$U_{\text{sc}}\left(r_{ij}\right)$ was designed to allow the passage of the chromatin chain by itself with an energetic barrier of E_{cut}. It mimicked the effect of enzymes, such as topoisomerase II, which allow chain crossing by cutting and reannealing the polymer[3].

We further confined chromatin in a sphere to mimic the effect of chromosome territories with the potential U_{confine} , defined as follows:

$$U_{\text{confine}} = \sum_{i=1}^{N} \max\left(0, \kappa(\|\vec{r_i}\| - R)^2\right)$$

where $\kappa = 1000k_B T$ and the radius R is set to ensure a nucleosome concentration of 0.2mM. $\|\cdot\|$ represents the L2 norm of the vector.

The last term accounts for the sequence dependent interactions derived from the condense-seq experiments, defined as follows:

$$U_{\text{contact}} = \sum_{i>j}^{N} \frac{\epsilon_0 |j-i|^{\alpha} + \epsilon_{ij}}{2} \left(1 + \tanh\left(\mu\left(r_c - r_{ij}\right)\right)\right),$$

where $r_c = 1.42\sigma$ and $\mu = \frac{4}{\sigma}$. The pre-factor $\epsilon_0|j-i|^{\alpha} + \epsilon_{ij}$ determines the depth of the potential. Here, there are two separate effects captured. The first term, $\epsilon_0|j-i|^{\alpha}$, is a separation dependent term that aims to capture the power law relationship of genomic contacts characteristic of Hi-C contact maps. This adjustment term is necessary because of the significant coarse-graining on the nucleosomes. We used $\epsilon_0 = -0.5$ and $\alpha = -0.5$.

The second term in the pre-factor, ϵ_{ij}, is the parameter derived from condense-seq experiments using phase separation theory [unpublished]. This term is defined as follows:

$$\epsilon_{ij} = -\beta\left(\varphi_i \varphi_j - \bar{\varphi}^2\right)$$

where $\beta = 0.6$ and $\bar{\varphi}$ is a constant to center the interaction energies, which was empirically determined to be 0.6. $\vec{\varphi}$ is a normalized and shifted vector of the read counts in the condensate obtained in the condense-seq experiments. Let $\vec{p}$ be the vector of read counts for each genomic segment in the condensate (i.e., the difference between read counts in the input control and supernatant samples).

$$\vec{q} = \frac{\vec{p} - \min \vec{p}}{\max \vec{p} - \min \vec{p}}$$

$$\vec{\varphi} = \vec{q} - \langle \vec{q} \rangle + \bar{\varphi}$$

We carried out coarse-grained molecular dynamics simulations of chromatin using OpenMM software[4]. Sixteen independent trajectories were simulated using a Langevin integrator. Each trajectory was first initialized by placing the beads along a fractal 3D Hilbert curve, and then the system was allowed to equilibrate for 10 million time steps at size 0.005. The equilibration energy function was identical to the simulation energy, except for the sequence dependent contact potential U_{contact} , which was set to zero. After equilibration, simulations were run for 80 million time steps at size $0.01t$, and snapshots were saved every 10,000 steps. Each trajectory thus provided 8,000 snapshots. The contact probabilities for these from 16 independent trajectories were averaged to obtain an average contact probability matrix of 128,000 total frames. Summary metrics such as compartment scores and TAD insulation boundaries, were computed from these contact matrixes and compared with the same metrics as in the experimental Hi-C maps.

Supplementary Note 3. Single molecule experiment protocol.

Sample preparation for single molecule FRET and pulldown

Biotinylated 20N0 DNA and fluorophore-labeled Cy3/Cy5 20N20 DNA were generated by PCR amplification (N stands for the Widom 601-c2 nucleosome positioning sequence, and 20 or 0 represent the linker DNA length in bp on either end). Primers were purchased from Integrated DNA Technologies Inc. (IDT). Oligos containing amine-modifications were labeled by mixing 8mM NHS-dye (Lumiprobe), 160uM oligo, and 200mM fresh NaHCO3 for 4 hours at room temperature (RT), followed by an overnight incubation at 4°C. Free dye was removed using ethanol precipitation. Labeling efficiency was determined to be over 90% for all constructs using Nanodrop. PCR reactions (2.5ml volume) were performed using Phusion High-Fidelity PCR Master Mix (New England Biolabs). Nucleosomal DNA was purified over a 6% polyacrylamide 29:1 Native PAGE column (Bio-Rad Mini Prep Cell) using 0.5X TBE buffer (300V for 4 hours at RT). Fractions were identified using 2% agarose gel, concentrated and stored at -20°C. Full DNA sequence information can be found in Supplementary Table 12. Nucleosomes were similarly reconstituted as previously mentioned in the method section of main text. Human histone octamer was used to reconstitute Cy3/Cy5 20N20 mononucleosomes. 20N0 mononucleosomes were reconstituted using an octamer containing histones from two different organisms: Cy3-H2A(K120C) and H2B from Saccharomyces cerevisiae, H3 and H4 from Drosophila melanogaster. Lambda DNA was added as part of the reconstitution reaction to prevent excess histone binding to linker DNA or aggregation. Mononucleosomes were purified over 5-20% sucrose gradient. After purification, nucleosome containing fractions were concentrated and dialyzed into low-salt buffer (10 mM tris at pH 7.5, 50 mM NaCl, 1 mM EDTA, 3.5 mM BME, and 0.02% NP-40) overnight at 4°C. Nucleosomes were stored by adding 100% sterile filtered glycerol to a final concentration of 20%.

Single molecule imaging instrumentation and preparation

A prism-based total internal reflection fluorescence (TIRF) microscope was used for single-molecule imaging on a Nikon Eclipse Ti microscope. Solid-state lasers 641nm (Coherent) and 543nm (Shanghai Dream Lasers Technology) were used for illumination. A water immersed 60x/1.27 NA objective was used to collect emission and fluorescence was sorted to two channels using a custom laser-blocking filter and dichroic mirrors. Parameters for spot detection and FRET efficiency calculations (donor bleed-through, background correction and Cy3-Cy5 crosstalk correction) were determined as discussed previously[5]. Data analysis was performed using custom IDL (Interactive Data Language) and MATLAB scripts (https://sites.google.com/site/taekjiphalab/resources). Quartz slides and glass coverslips passivated with methoxy polyethylene glucose (PEG) and biotin-(PEG) were prepared using previously described methods[6] and assembled into flow chambers. Neutravidin (0.2mg/ml in 10mM Tris-HCl pH 8.0 and 50mM NaCl) was flown into each channel to functionalize the surface. The neutravidin solution was washed off and the chamber was immediately used for nucleosome immobilization.

Supplementary Note 4. Additional interpretation of condense-seq data.

The correlation between condensability and AT content of DNA

We could see a clear correlation between nucleosome condensability and AT content of DNA in many data sets, such as the simple correlation (Extended Data Fig. 5a), conditional correlation (Fig. 3 b), boosting analysis (new Extended Data Fig. 5h), the meta-gene profile near TSS (Fig. 1e). However, these correlations do not mean that AT content alone determines the nucleosome condensability. As shown by the strong cell type dependence (Fig. 1f), DNA sequence cannot be a sole determinant of condensability. In addition, the condense-seq data with reconstituted nucleosome clearly show the primary importance of PTMs in determining nucleosome condensability (Extended Data Fig. 7). The AT content dependence is at least in part due to the confounding effects of various genetic and epigenetic factors since genomic nucleosomes with low AT content tend to be more highly decorated with acetylation and vice-versa (Extended Data Fig. 5e).

The evidence supporting the electrostatic nature of nucleosome condensation

Our condense-seq data using purified native genomic nucleosomes and synthetic histone PTM library using a variety of condensing agents suggest that electrostatic interactions appear to be a major determinant for nucleosome condensability for the following reasons.

(1) In the synthetic histone PTM library data (Fig. 3d-f), acetylation has the largest effect of decreasing condensation among all possible modifications (except ubiquitylation) and, especially, poly-acetylation gives the strongest effects. This strong acetylation dependency trend is also further consistent with our mass spectrometry data (Extended Data Fig 6a,b) and conditional correlation analysis on genomic nucleosome (Fig. 3b). Because acetylation removes a positive charge, our data support the importance of electrostatic interactions in determining nucleosome condensability.

(2) The acidic patch mutations that reverse the charge from negative to positive of the histone core greatly increase the condensability ("H2A/H2B AP mutant" in tabular bar plot of Fig. 3e), further supporting the electrostatic basis for nucleosome condensability.

(3) Condensability scores are cross–correlated with each other when we used different polyamines (spermine vs spermidine) or non-polyamine-based multivalent cations such as Cobalt hexamine (Fig. 3a, Extended Data Fig 8b, and Extended Data Fig. 9f), which are condensing agents known for charge-charge interaction-based mechanism. All this data supports the idea that electrostatics, which is a ubiquitous force regardless of the identity of protein interactors, is a major determinant of nucleosome condensability.

Combined with our observation that polymer simulations using nucleosome condensability as the sole input can reproduce the large-scale genome organization into A and B compartments, our data indicate that the electrostatic character of the mono-nucleosome determines nucleosome condensability and thereby the formation of A/B compartments.

Supplementary Table 1. Condense-seq data.

Name	Cell	Sample type	Condensing agent	Sample ID#
**H1_NCP_sp_0**	H1 hESC	NCP	spermine (4+)	0
**H1_NCP_sp_1**	H1 hESC	NCP	spermine (4+)	1
**H1_NCP_sp_2**	H1 hESC	NCP	spermine (4+)	2
**H1_NCP_sp_3**	H1 hESC	NCP	spermine (4+)	3
**H1_NCP_sp_4**	H1 hESC	NCP	spermine (4+)	4
**H1_NCP_sp_5**	H1 hESC	NCP	spermine (4+)	5
**H1_NCP_sp_6**	H1 hESC	NCP	spermine (4+)	6
**H1_NCP_sp_7**	H1 hESC	NCP	spermine (4+)	7
**H1_NCP_sp_8**	H1 hESC	NCP	spermine (4+)	8
**H1_NCP_sp_9**	H1 hESC	NCP	spermine (4+)	9
**H1_NCP_spd_0**	H1 hESC	NCP	spermidine (3+)	0
**H1_NCP_spd_6**	H1 hESC	NCP	spermidine (3+)	6
**H1_NCP_CoH_0**	H1 hESC	NCP	Cobalt Hexammine (3+)	0
**H1_NCP_CoH_5**	H1 hESC	NCP	Cobalt Hexammine (3+)	5
**H1_NCP_PEG_0**	H1 hESC	NCP	PEG 8000	0
**H1_NCP_PEG_6**	H1 hESC	NCP	PEG 8000	6
**H1_NCP_Ca_0**	H1 hESC	NCP	Ca (2+)	0
**H1_NCP_Ca_5**	H1 hESC	NCP	Ca (2+)	5
**H1_NCP_HP1a_0**	H1 hESC	NCP	HP1 alpha	0
**H1_NCP_HP1a_1**	H1 hESC	NCP	HP1 alpha	1
**H1_NCP_HP1a_2**	H1 hESC	NCP	HP1 alpha	2
**H1_NCP_HP1a_3**	H1 hESC	NCP	HP1 alpha	3
**H1_NCP_HP1a_4**	H1 hESC	NCP	HP1 alpha	4
**H1_NCP_HP1a_5**	H1 hESC	NCP	HP1 alpha	5
**H1_NCP_HP1bSUV_0**	H1 hESC	NCP	HP1 beta + tSUV39h1	0
**H1_NCP_HP1bSUV_1**	H1 hESC	NCP	HP1 beta + tSUV39h1	1
**H1_NCP_HP1bSUV_2**	H1 hESC	NCP	HP1 beta + tSUV39h1	2
**H1_NCP_HP1bSUV_3**	H1 hESC	NCP	HP1 beta + tSUV39h1	3
**H1_NCP_HP1bSUV_4**	H1 hESC	NCP	HP1 beta + tSUV39h1	4
**H1_NCP_HP1bSUV_5**	H1 hESC	NCP	HP1 beta + tSUV39h1	5
**H1_DNA_HP1a_0**	H1 hESC	DNA	HP1 alpha	0
**H1_DNA_HP1a_3**	H1 hESC	DNA	HP1 alpha	3
**GM_NCP_sp_0**	GM12878	NCP	spermine (4+)	0
**GM_NCP_sp_1**	GM12878	NCP	spermine (4+)	1
**GM_NCP_sp_2**	GM12878	NCP	spermine (4+)	2
**GM_NCP_sp_3**	GM12878	NCP	spermine (4+)	3

**GM_NCP_sp_4**	GM12878	NCP	spermine (4+)	4
**GM_NCP_sp_5**	GM12878	NCP	spermine (4+)	5
**GM_NCP_sp_6**	GM12878	NCP	spermine (4+)	6
**GM_NCP_sp_7**	GM12878	NCP	spermine (4+)	7
**GM_NCP_sp_8**	GM12878	NCP	spermine (4+)	8
**GM_NCP_sp_9**	GM12878	NCP	spermine (4+)	9
**mCD8T:WT_NCP_sp_0**	mouse CD8 T cell (WT)	NCP	spermine (4+)	0
**mCD8T:WT_NCP_sp_1**	mouse CD8 T cell (WT)	NCP	spermine (4+)	1
**mCD8T:WT_NCP_sp_2**	mouse CD8 T cell (WT)	NCP	spermine (4+)	2
**mCD8T:WT_NCP_sp_3**	mouse CD8 T cell (WT)	NCP	spermine (4+)	3
**mCD8T:WT_NCP_sp_4**	mouse CD8 T cell (WT)	NCP	spermine (4+)	4
**mCD8T:WT_NCP_sp_5**	mouse CD8 T cell (WT)	NCP	spermine (4+)	5
**mCD8T:WT_NCP_sp_6**	mouse CD8 T cell (WT)	NCP	spermine (4+)	6
**mCD8T:WT_NCP_sp_7**	mouse CD8 T cell (WT)	NCP	spermine (4+)	7
**mCD8T:WT_NCP_sp_8**	mouse CD8 T cell (WT)	NCP	spermine (4+)	8
**mCD8T:WT_NCP_sp_9**	mouse CD8 T cell (WT)	NCP	spermine (4+)	9
**mCD8T:DFMO_NCP_sp_0**	mouse CD8 T cell (+DFMO)	NCP	spermine (4+)	0
**mCD8T:DFMO_NCP_sp_1**	mouse CD8 T cell (+DFMO)	NCP	spermine (4+)	1
**mCD8T:DFMO_NCP_sp_2**	mouse CD8 T cell (+DFMO)	NCP	spermine (4+)	2
**mCD8T:DFMO_NCP_sp_3**	mouse CD8 T cell (+DFMO)	NCP	spermine (4+)	3
**mCD8T:DFMO_NCP_sp_4**	mouse CD8 T cell (+DFMO)	NCP	spermine (4+)	4
**mCD8T:DFMO_NCP_sp_5**	mouse CD8 T cell (+DFMO)	NCP	spermine (4+)	5
**mCD8T:DFMO_NCP_sp_6**	mouse CD8 T cell (+DFMO)	NCP	spermine (4+)	6
**mCD8T:DFMO_NCP_sp_7**	mouse CD8 T cell (+DFMO)	NCP	spermine (4+)	7
**mCD8T:DFMO_NCP_sp_8**	mouse CD8 T cell (+DFMO)	NCP	spermine (4+)	8
**mCD8T:DFMO_NCP_sp_9**	mouse CD8 T cell (+DFMO)	NCP	spermine (4+)	9
**mCD8T:ODCKO_NCP_sp_0**	mouse CD8 T cell (ODC KO)	NCP	spermine (4+)	0
**mCD8T:ODCKO_NCP_sp_1**	mouse CD8 T cell (ODC KO)	NCP	spermine (4+)	1
**mCD8T:ODCKO_NCP_sp_2**	mouse CD8 T cell (ODC KO)	NCP	spermine (4+)	2
**mCD8T:ODCKO_NCP_sp_3**	mouse CD8 T cell (ODC KO)	NCP	spermine (4+)	3
**mCD8T:ODCKO_NCP_sp_4**	mouse CD8 T cell (ODC KO)	NCP	spermine (4+)	4
**mCD8T:ODCKO_NCP_sp_5**	mouse CD8 T cell (ODC KO)	NCP	spermine (4+)	5
**mCD8T:ODCKO_NCP_sp_6**	mouse CD8 T cell (ODC KO)	NCP	spermine (4+)	6
**mCD8T:ODCKO_NCP_sp_7**	mouse CD8 T cell (ODC KO)	NCP	spermine (4+)	7
**mCD8T:ODCKO_NCP_sp_8**	mouse CD8 T cell (ODC KO)	NCP	spermine (4+)	8
**mCD8T:ODCKO_NCP_sp_9**	mouse CD8 T cell (ODC KO)	NCP	spermine (4+)	9
**GM_synNCP_sp_0**	GM12878	Reconstituted NCP	spermine (4+)	0
**GM_synNCP_sp_1**	GM12878	Reconstituted NCP	spermine (4+)	1

**GM_synNCP_sp_2**	GM12878	Reconstituted NCP	spermine (4+)	2
**GM_synNCP_sp_3**	GM12878	Reconstituted NCP	spermine (4+)	3
**GM_synNCP_sp_4**	GM12878	Reconstituted NCP	spermine (4+)	4
**GM_synNCP_sp_5**	GM12878	Reconstituted NCP	spermine (4+)	5
**GM_synNCP_sp_6**	GM12878	Reconstituted NCP	spermine (4+)	6
**GM_synNCP_sp_7**	GM12878	Reconstituted NCP	spermine (4+)	7
**GM_synNCP_sp_8**	GM12878	Reconstituted NCP	spermine (4+)	8
**GM_synNCP_sp_9**	GM12878	Reconstituted NCP	spermine (4+)	9
**E14_NCP_sp_0**	E14 mESC	NCP	spermine (4+)	0
**E14_NCP_sp_1**	E14 mESC	NCP	spermine (4+)	1
**E14_NCP_sp_2**	E14 mESC	NCP	spermine (4+)	2
**E14_NCP_sp_3**	E14 mESC	NCP	spermine (4+)	3
**E14_NCP_sp_4**	E14 mESC	NCP	spermine (4+)	4
**E14_NCP_sp_5**	E14 mESC	NCP	spermine (4+)	5
**E14_NCP_sp_6**	E14 mESC	NCP	spermine (4+)	6
**E14_NCP_sp_7**	E14 mESC	NCP	spermine (4+)	7
**E14_NCP_sp_8**	E14 mESC	NCP	spermine (4+)	8
**E14_NCP_sp_9**	E14 mESC	NCP	spermine (4+)	9

Supplementary Table 2. Histone ChIP-seq data used for human cell lines.

Name	Cell	Source	BAM data files	BAM control files	Bed files
**H3K79me2**	H1-hESC	Bradley Bernstein, Broad, ENCODE	ENCFF334FBV, ENCFF965LBC	ENCFF064DDT, ENCFF504ZFN	ENCFF344MEX
**H3K27ac**	H1-hESC	Bradley Bernstein, Broad, ENCODE	ENCFF238SQN, ENCFF242PAC	ENCFF064DDT, ENCFF504ZFN	ENCFF317QGQ
**H3K4me3**	H1-hESC	Bradley Bernstein, Broad, ENCODE	ENCFF775QSF, ENCFF262WSA	ENCFF064DDT, ENCFF504ZFN	ENCFF668YOE
**H3K9ac**	H1-hESC	Bradley Bernstein, Broad, ENCODE	ENCFF707RSH, ENCFF101FLH	ENCFF064DDT, ENCFF504ZFN	ENCFF436XTS
**H4K20me1**	H1-hESC	Bradley Bernstein, Broad, ENCODE	ENCFF890EPF, ENCFF767VBX	ENCFF064DDT, ENCFF504ZFN	ENCFF997CKL
**H3K4me1**	H1-hESC	Bradley Bernstein, Broad, ENCODE	ENCFF818MAU, ENCFF052LQE	ENCFF064DDT, ENCFF504ZFN	ENCFF238YJA
**H3K9me3**	H1-hESC	Bradley Bernstein, Broad, ENCODE	ENCFF669PQL, ENCFF230VQG	ENCFF064DDT, ENCFF504ZFN	ENCFF918VFL

**H3K4me2**	H1-hESC	Bradley Bernstein, Broad, ENCODE	ENCFF694EXK, ENCFF115ZGZ	ENCFF064DDT, ENCFF504ZFN	ENCFF836LZM
**H3K27me3**	H1-hESC	Bradley Bernstein, Broad, ENCODE	ENCFF270IOE, ENCFF171RVK	ENCFF064DDT, ENCFF504ZFN	ENCFF296RYM
**H3K36me3**	H1-hESC	Bradley Bernstein, Broad, ENCODE	ENCFF247BVI, ENCFF204EVH	ENCFF064DDT, ENCFF504ZFN	ENCFF813VFV
**H2AFZ**	H1-hESC	Bradley Bernstein, Broad, ENCODE	ENCFF673SFE, ENCFF156BHJ	ENCFF064DDT, ENCFF504ZFN	ENCFF745GKP
**H2BK20ac**	H1-hESC	Bing Ren, UCSD, Roadmap	ENCFF115TTE, ENCFF287BTK	ENCFF110QNK, ENCFF393RKZ	ENCFF215EPH
**H3K14ac**	H1-hESC	Bing Ren, UCSD, Roadmap	ENCFF278FDJ, ENCFF474FQS	ENCFF899CRH	ENCFF870OAL
**H2AK5ac**	H1-hESC	Bing Ren, UCSD, Roadmap	ENCFF343MLC, ENCFF282MTU	ENCFF110QNK, ENCFF956GEE	ENCFF320JIP
**H4K5ac**	H1-hESC	Bing Ren, UCSD, Roadmap	ENCFF564KDZ, ENCFF159YCB	ENCFF572GEP,E NCFF899CRH	ENCFF097DDN
**H3K79me1**	H1-hESC	Bing Ren, UCSD, Roadmap	ENCFF668IKK, ENCFF679WYW	ENCFF393RKZ, ENCFF236MNB	ENCFF088PTH
**H3K56ac**	H1-hESC	Bing Ren, UCSD, Roadmap	ENCFF465SCK, ENCFF667SPV	ENCFF110QNK, ENCFF899CRH	ENCFF600YNW
**H3K18ac**	H1-hESC	Bing Ren, UCSD, Roadmap	ENCFF169AXD, ENCFF830BDG	ENCFF572GEP, ENCFF728FDB	ENCFF192GAX
**H3K4ac**	H1-hESC	Bing Ren, UCSD, Roadmap	ENCFF763OXL, ENCFF556XYF	ENCFF110QNK, ENCFF899CRH	ENCFF711LQB
**H2BK120ac**	H1-hESC	Bing Ren, UCSD, Roadmap	ENCFF669EZB, ENCFF960RNZ	ENCFF110QNK, ENCFF899CRH	ENCFF658MQQ
**H2BK12ac**	H1-hESC	Bing Ren, UCSD, Roadmap	ENCFF207RMW, ENCFF989LMR	ENCFF110QNK, ENCFF956GEE	ENCFF237XZS
**H4K8ac**	H1-hESC	Bing Ren, UCSD, Roadmap	ENCFF349SRS, ENCFF293PPG	ENCFF899CRH, ENCFF956GEE	ENCFF760EFQ
**H3K23ac**	H1-hESC	Bing Ren, UCSD, Roadmap	ENCFF334PGH, ENCFF457BIS	ENCFF236MNB, ENCFF899CRH	ENCFF350XJF
**H2BK15ac**	H1-hESC	Bing Ren, UCSD, Roadmap	ENCFF911CPW, ENCFF659DKC	ENCFF393RKZ, ENCFF728FDB	ENCFF855ILJ
**H4K91ac**	H1-hESC	Bing Ren, UCSD, Roadmap	ENCFF710IHP, ENCFF466YRA	ENCFF110QNK, ENCFF899CRH	ENCFF964FVB

**H2BK5ac**	H1-hESC	Bing Ren, UCSD, Roadmap	ENCFF458NUT, ENCFF821ZBS	ENCFF110QNK, ENCFF956GEE	ENCFF929FTR
**H3K23me2**	H1-hESC	Bing Ren, UCSD, Roadmap	ENCFF415JDK, ENCFF199NJK	ENCFF236MNB, ENCFF728FDB	ENCFF567WXW
**H3K9me3**	GM12878	Bradley Bernstein, Broad, ENCODE	ENCFF663EWP, ENCFF758GUH	ENCFF797ARJ, ENCFF873ZWP	ENCFF725UFY
**H3K36me3**	GM12878	Bradley Bernstein, Broad, ENCODE	ENCFF926HQH, ENCFF297QVO	ENCFF559AEN, ENCFF908JTQ	ENCFF432EMI
**H4K20me1**	GM12878	Bradley Bernstein, Broad, ENCODE	ENCFF880XJW, ENCFF937PBY	ENCFF873ZWP, ENCFF797ARJ	ENCFF469QYW
**H3K4me1**	GM12878	Bradley Bernstein, Broad, ENCODE	ENCFF153KPG, ENCFF815TLX	ENCFF873ZWP, ENCFF797ARJ	ENCFF321BVG
**H3K4me2**	GM12878	Bradley Bernstein, Broad, ENCODE	ENCFF803ROB, ENCFF128WUO	ENCFF684NLR, ENCFF651UIO	ENCFF283LNH
**H3K4me3**	GM12878	Bradley Bernstein, Broad, ENCODE	ENCFF396LGW, ENCFF634CBL	ENCFF142VHH, ENCFF401XXH	ENCFF998CEU
**H3K27ac**	GM12878	Bradley Bernstein, Broad, ENCODE	ENCFF804NCH, ENCFF948GTC	ENCFF651UIO, ENCFF684NLR	ENCFF023LTU
**H3K9ac**	GM12878	Bradley Bernstein, Broad, ENCODE	ENCFF737GSB, ENCFF424IMO	ENCFF797ARJ, ENCFF873ZWP	ENCFF981JOU
**H3K27me3**	GM12878	Bradley Bernstein, Broad, ENCODE	ENCFF231DJN, ENCFF175YYN	ENCFF651UIO, ENCFF684NLR	ENCFF291DHI
**H3K79me2**	GM12878	Bradley Bernstein, Broad, ENCODE	ENCFF676NDU, ENCFF231YZJ	ENCFF797ARJ, ENCFF873ZWP	ENCFF131ZGZ
**H2AFZ**	GM12878	Bradley Bernstein, Broad, ENCODE	ENCFF762TRA, ENCFF848PUT	ENCFF797ARJ, ENCFF873ZWP	ENCFF377OJG

Supplementary Table 3. Bisulfite-seq data used for human cell lines.

Name	Cell	Source	BAM data files	BAM control files	Bed files
**CpG**	H1-hESC	Richard Myers, HAIB	NA	NA	ENCFF434CNG
**CHH**	H1-hESC	Richard Myers, HAIB	NA	NA	ENCFF169UFF
**CHG**	H1-hESC	Richard Myers, HAIB	NA	NA	ENCFF780ECA
**CpG**	GM12878	Richard Myers, HAIB	NA	NA	ENCFF570TIL
**CHH**	GM12878	Richard Myers, HAIB	NA	NA	ENCFF187KAK

**CHG**	GM12878	Richard Myers, HAIB	NA	NA	ENCFF910HOG

Supplementary Table 4. RNA-seq data used for human cell lines.

Name	Cell	Source	BAM data files	BAM control files	tsv files
**Gene-quantification**	H1-hESC	Barbara Wold, Caltech	NA	NA	ENCFF174OMR
**Gene-quantification**	GM12878	Barbara Wold, Caltech	NA	NA	ENCFF345SHY

Supplementary Table 5. Transcription factors ChIP-seq data used for human cell lines.

Name	Cell	Source	BAM data files	BAM control files	Bed files
**CTCF**	H1-hESC	Bradley Bernstein, Broad	ENCFF740VTK	ENCFF064DDT	ENCFF692RPA
**CBX5**	H1-hESC	Bradley Bernstein, Broad	NA	NA	ENCFF218OXB
**RNF2**	H1-hESC	Bradley Bernstein, Broad	NA	NA	ENCFF241UKW
**CBX8**	H1-hESC	Bradley Bernstein, Broad	NA	NA	ENCFF483UZG
**SUZ12**	H1-hESC	Bradley Bernstein, Broad	NA	NA	ENCFF233GVJ
**EZH2**	H1-hESC	Bradley Bernstein, Broad	NA	NA	ENCFF414CAB
**POLR2A**	H1-hESC	Richard Myers, HAIB	NA	NA	ENCFF322DAE
**POLR2AphosphoS5**	H1-hESC	Richard Myers, HAIB	NA	NA	ENCFF872MKT
**Rad21**	H1-hESC	Michael Snyder, Stanford	NA	NA	ENCFF883FUW
**CTCF**	GM12878	Michael Snyder, Stanford	ENCFF162QXM, ENCFF033WII	ENCFF157YWH	ENCFF796WRU

Supplementary Table 6. 3D genomic data used for human cell lines.

Name	Cell	Assay	File type	Processed data
**Micro-C**	H1-hESC	Micro-C	hic	4DNFI2TK7L2F
**SON**	H1-hESC	TSA-seq	bw	4DNFI625PP2A, 4DNFIFKMOD1L
**LaminB1**	H1-hESC	DamID-seq	bw	4DNFIXNBG8L1

Supplementary Table 7. Additional 3D genomic data used for human cell lines.

Name	Cell	Assay	Source	File type	Processed data
**Nucleolar**	K562	DamID-seq	GSE148609	bw	GSE148609_K562_4xAP3-50kb-combined.bw
**Ki67**	hTERT RPE-1, HCT116, K562	DamID-seq	GSE186206	tsv	GSE186206_pADamID_combined.tsv.gz

Supplementary Table 8. Histone ChIP-seq data used for mouse cell lines.

Name	Cell	Source	Data files (FASTQ or BAM)	Control files (FASTQ or BAM)	Processing pipeline
**H3K4me1**	Mouse CD8 T cell (invitro activated)	GSE54191	SRR1124802, SRR1124803	SRR1124815, SRR1124816, SRR1124817	ENCODE
**H3K4me3**	Mouse CD8 T cell (invitro activated)	GSE54191	SRR1124804, SRR1124805	SRR1124815, SRR1124816, SRR1124817	ENCODE
**H3K27ac**	Mouse CD8 T cell (invitro activated)	GSE54191	SRR1124806, SRR1124807	SRR1124815, SRR1124816, SRR1124817	ENCODE
**H3K27me3**	Mouse CD8 T cell (invitro activated)	GSE54191	SRR1124808, SRR1124809	SRR1124815, SRR1124816, SRR1124817	ENCODE
**H3K36me3**	Mouse CD8 T cell (invitro activated)	GSE54191	SRR1124810, SRR1124811	SRR1124815, SRR1124816, SRR1124817	ENCODE
**H3K9me3**	Mouse CD8 T cell (invitro activated)	GSE106265	SRR6228885	SRR6228886	ENCODE
**H3K27me3**	Mouse embryonic stem cell (Smarca4V5_WT)	GSE77093	SRR3111667, SRR3111668	SRR3111706	ENCODE
**H3K4me1**	E14TG2a.4	ENCSR032JUI	ENCFF975ODF, ENCFF723RHZ	ENCFF984AYM, ENCFF826KVZ	ENCODE

**H3K4me3**	E14TG2a.4	ENCSR212KGS	ENCFF537HSV, ENCFF151MSO	ENCFF984AYM, ENCFF826KVZ	ENCODE
**H3K27me3**	E14TG2a.4	ENCSR059MBO	ENCFF655DRS, ENCFF891APM	ENCFF984AYM, ENCFF826KVZ	ENCODE
**H3K36me3**	E14TG2a.4	ENCSR253QPK	ENCFF237WSM, ENCFF746CGG	ENCFF984AYM, ENCFF826KVZ	ENCODE
**H3K9me3**	E14TG2a.4	ENCSR857MYS	ENCFF293HOI, ENCFF198KZO	ENCFF984AYM, ENCFF826KVZ	ENCODE
**H3K27ac**	ES-E14	ENCSR000CGQ	ENCFF542DVQ, ENCFF477QZK	ENCFF083BUU, ENCFF664MLQ	ENCODE
**H3K9ac**	ES-E14	ENCSR000CGP	ENCFF289ZJV, ENCFF405QYS	ENCFF083BUU, ENCFF664MLQ	ENCODE

Supplementary Table 9. RNA-seq data used for mouse cell lines.

Name	Cell	Source	FASTQ data files	Read count files
**RNA-seq**	Mouse CD8 T cell (invitro activated)	GSE129772	RR8893037, SRR8893038, SRR8893039	GSM3721901, GSM3721902, GSM3721903
**RNA-seq**	Mouse CD8 T cell (invitro activated)	GSE199666	SRR18517477, SRR18517476, SRR18517475	GSM5980043, GSM5980044, GSM5980045
**RNA-seq**	Mouse CD8 T cell (invitro activated)	GSE136905	SRR10070511, SRR10070512	GSM4061343, GSM4061344
**RNA-seq**	ES-E14	ENCSR000CWC	NA	ENCFF827OZU

Supplementary Table 10. 3D genomic data used for mouse cell lines.

Name	Cell	Source	FASTQ files	Processing pipeline
**Hi-C**	Mouse CD8 T cell (invitro activated)	GSE158375	SRR12693619	4D nucleome

Supplementary Table 11. Gene annotation data table.

Organism	Version	GTF file

**Human**	GENCODE V29	ENCFF159KBI
**Mouse**	GENCODE VM21	ENCFF871VGR

Supplementary Table 12. DNA sequence information of nucleosomes used in single molecule experiments.

Type of NCP	Primers used	Sequences	Fluorophores used	Type of experiment
20N20	Forward primer:<br>GAATTCGCCCTTGCCTGCAGacaggatgtatatatcttacacgtgcctagagactagtaagtaatccta/iAmMC6T/tggcgg<br>Reverse primer:<br>/5Biosg/GAATTCGCCCTTGCATCGATctggagatacc/iAmMC6T/ggtgctaaggccgct	Biotin-GAATTCGCCCTTGCATCGATctggagatacctggtgctaaggccgcttaattggtcgtagcaagctctagcaccgcttaaacgcacgtacgcgctgtctaccgcgttttaaccgccaataggattacttactagtctctaggcacgtgtaagatatatacatcctgtCTGCAGGCAAGGGCGAATTC	Cy3/Cy5	DNA unwrapping
20N0	Forward primer:<br>/5Biosg/GA ATT CGC CCT TGC CTG CAG<br>Reverse primer:<br>CTG GAG ATA CCC GGT GCT AAG	Biotin-GAATTCGCCCTTGCCTGCAGacaggatgtatatatcttacacgtgcctagagactagtaagtaatcctattggcggttaaaacgcggtagacagcgcgtacgtgcgtttaagcggtgctagagcttgctacgaccaattaagcggccttagcaccgggtatctccag	Cy3-H2A(K120C)	Nucleosome pulldown

Fig. 1b NCP

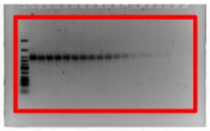

Fig. 1b DNA

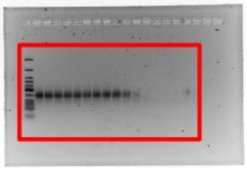

Extended Data Fig. 1a

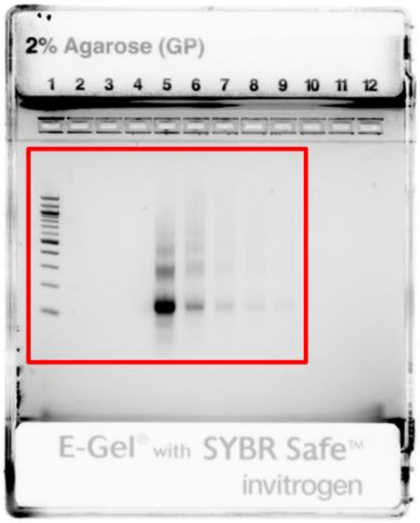

Extended Data Fig. 1b

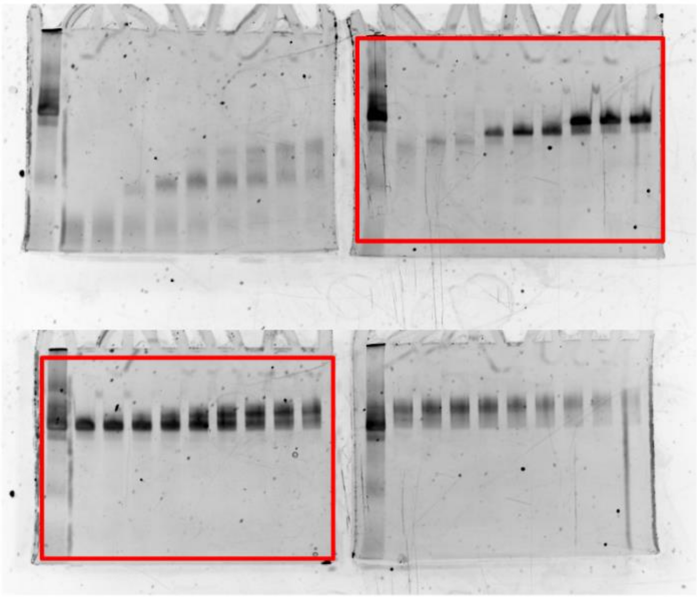

Extended Data Fig. 1c

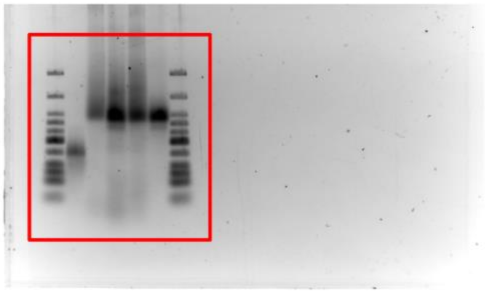

Extended Data Fig. 1d

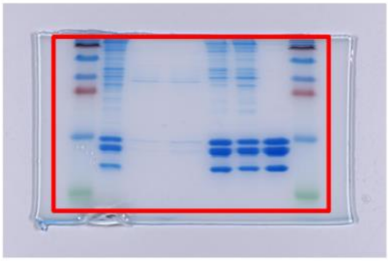

Supplementary Figure 1 | Uncropped gels (continued)

Extended Data Fig. 1e

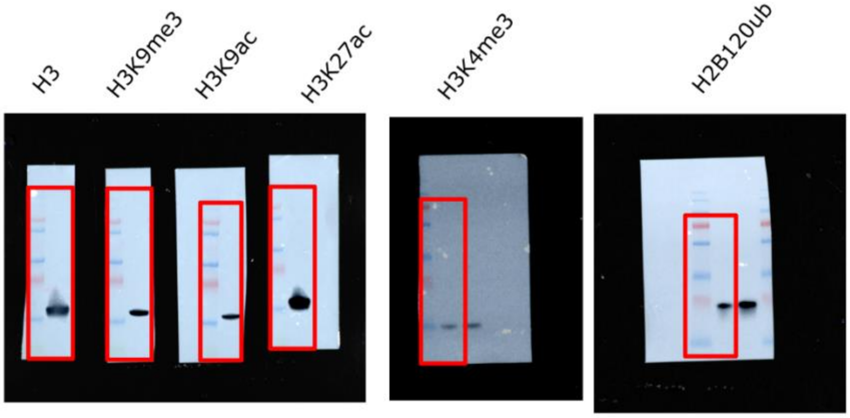

Extended Data Fig. 1h

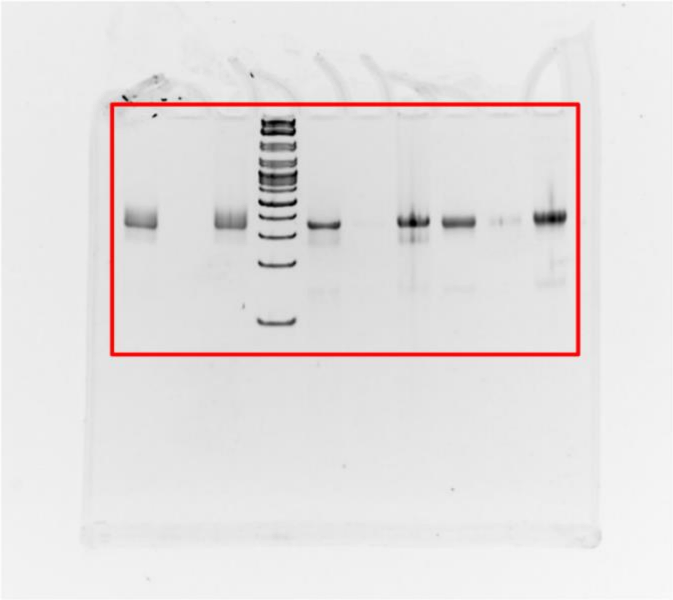

Extended Data Fig. 3d

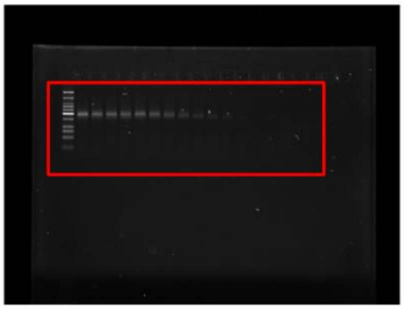

Extended Data Fig. 7b

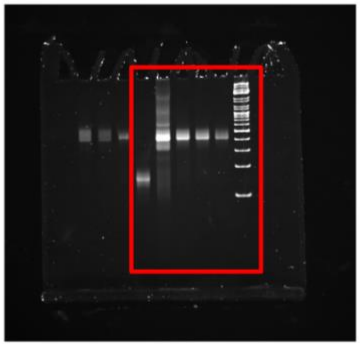

Extended Data Fig. 7c   Reconstituted NCP

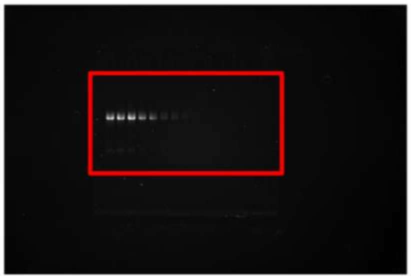

Extended Data Fig. 7c   Native NCP

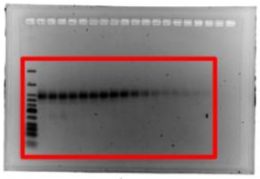

Supplementary Figure 1 | Uncropped gels (continued)

Extended Data Fig. 8a

NCP Spermidine^{3+}

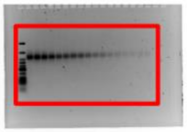

Extended Data Fig. 8a

DNA Spermidine^{3+}

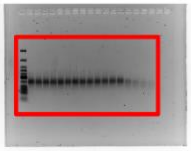

Extended Data Fig. 8a

NCP CoHex^{3+}

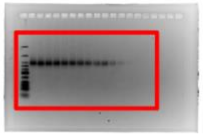

Extended Data Fig. 8a

DNA CoHex^{3+}

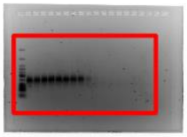

Extended Data Fig. 8a

NCP PEG 8000

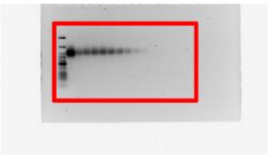

Extended Data Fig. 8a

DNA PEG 8000

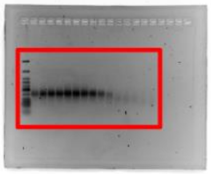

Extended Data Fig. 8a

NCP Mg^{2+}

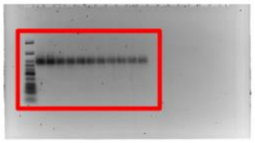

Extended Data Fig. 8a

DNA Ca^{2+}

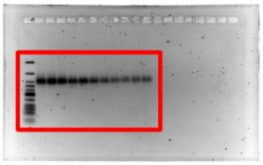

Extended Data Fig. 8a

NCP HP1α

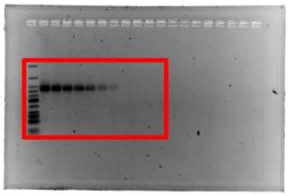

Extended Data Fig. 8a

DNA HP1α

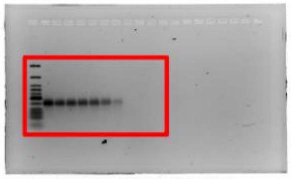

Extended Data Fig. 8a

NCP HP1β/SUV39H1

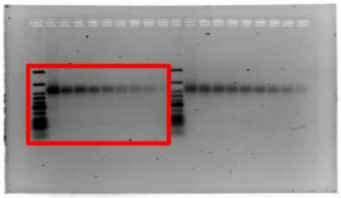

Extended Data Fig. 8a

DNA HP1β/SUV39H1

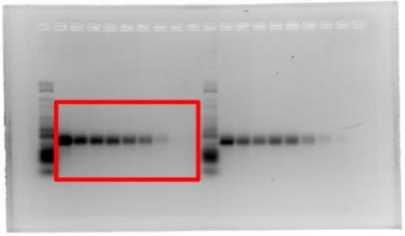

Supplementary Figure 1 | Uncropped gels (continued)

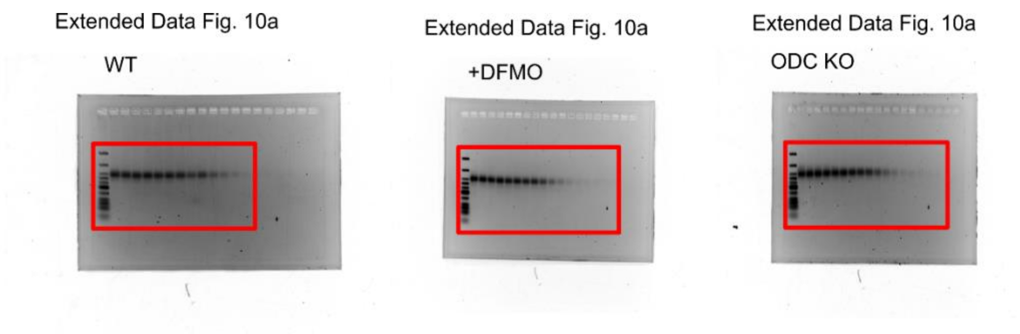

Supplementary Figure 1 | Uncropped gels

References

1 Reiff, S. B. *et al.* The 4D Nucleome Data Portal as a resource for searching and visualizing curated nucleomics data. *Nat Commun* **13**, 2365 (2022). https://doi.org/10.1038/s41467-022-29697-4

2 Brand, M., Rampalli, S., Chaturvedi, C. P. & Dilworth, F. J. Analysis of epigenetic modifications of chromatin at specific gene loci by native chromatin immunoprecipitation of nucleosomes isolated using hydroxyapatite chromatography. *Nat Protoc* **3**, 398-409 (2008). https://doi.org/10.1038/nprot.2008.8

3 Qi, Y. & Zhang, B. Predicting three-dimensional genome organization with chromatin states. *PLoS Comput Biol* **15**, e1007024 (2019). https://doi.org/10.1371/journal.pcbi.1007024

4 Eastman, P. *et al.* OpenMM 7: Rapid development of high performance algorithms for molecular dynamics. *PLoS Comput Biol* **13**, e1005659 (2017). https://doi.org/10.1371/journal.pcbi.1005659

5 Roy, R., Hohng, S. & Ha, T. A practical guide to single-molecule FRET. *Nat Methods* **5**, 507-516 (2008). https://doi.org/nmeth.1208 [pii]

10.1038/nmeth.1208

6 Paul, T. & Myong, S. Protocol for generation and regeneration of PEG-passivated slides for single-molecule measurements. *STAR Protoc* **3**, 101152 (2022). https://doi.org/10.1016/j.xpro.2022.101152
